# Genomic evidence of Oropouche virus autochthonous circulation in a small district in the state of Rio de Janeiro, Brazil

**DOI:** 10.1128/spectrum.02850-24

**Published:** 2025-02-11

**Authors:** Filipe Romero Rebello Moreira, Fábio Luís Lima Monteiro, Mariane Talon de Menezes, Pedro Junior Pinheiro Mourão, Camila de Almeida Velozo, Matheus Villanueva Andrade, Andréa Cony Cavalcanti, Mário Sérgio Ribeiro, Sílvia Cristina de Carvalho, Claudia Maria Braga de Mello, Rafael Mello Galliez, Terezinha Marta Pereira Pinto Castiñeiras, Fernanda de Bruycker Nogueira, Carolina Cardoso dos Santos, Ana Maria Bispo de Filippis, Átila Duque Rossi, Amilcar Tanuri, Carolina Moreira Voloch

**Affiliations:** 1 Departamento de Genética, Universidade Federal do Rio de Janeiro, Rio de Janeiro, Brazil; 2 Núcleo de Enfrentamento e Estudos de Doenças Infecciosas Emergentes e Reemergentes, Universidade Federal do Rio de Janeiro, Rio de Janeiro, Brazil; 3 Laboratório Central Noel Nutels, LACEN-RJ, Rio de Janeiro, Brazil; 4 Secretaria Estadual de Saúde, SES RJ, Rio de Janeiro, Brazil; 5 Departamento de Doenças Infecciosas e Parasitárias, Faculdade de Medicina, Universidade Federal do Rio de Janeiro, Rio de Janeiro, Brazil; 6 Laboratório de Flavivírus, Fundação Oswaldo Cruz, Rio de Janeiro, Brazil; University of Siena, Siena, Italy; Colorado State University, Fort Collins, Colorado, USA

**Keywords:** genomic epidemiology, phylogeography, emergence, virology

## LETTER

Oropouche virus (OROV; *Peribunyaviridae*), is an emerging arbovirus endemic to the Amazon Basin region of South America, which causes a febrile disease named Oropouche fever (1). This disease is usually mild and presents symptoms similar to other arboviral diseases, but more severe infections have been recently reported, including the first cases of abortion and fetal malformation due to vertical transmission and deaths in 2024 (2–7). This year marked the first OROV outbreak in Brazil outside the Amazon region, following successive resurgences in the endemic area (8). Genomic surveillance indicates that these outbreaks were caused by a reassortant OROV lineage, which presents the S, M, and L segments more closely related to distinct viral strains, including the OROV prototype, Iquitos, and Perdões viruses (9, 10). Preliminary experimental evidence suggests that this lineage replicates faster in mammalian cells and is less effectively neutralized by antibodies from prior infections compared to the prototype OROV strain (11).

**Fig 1:**
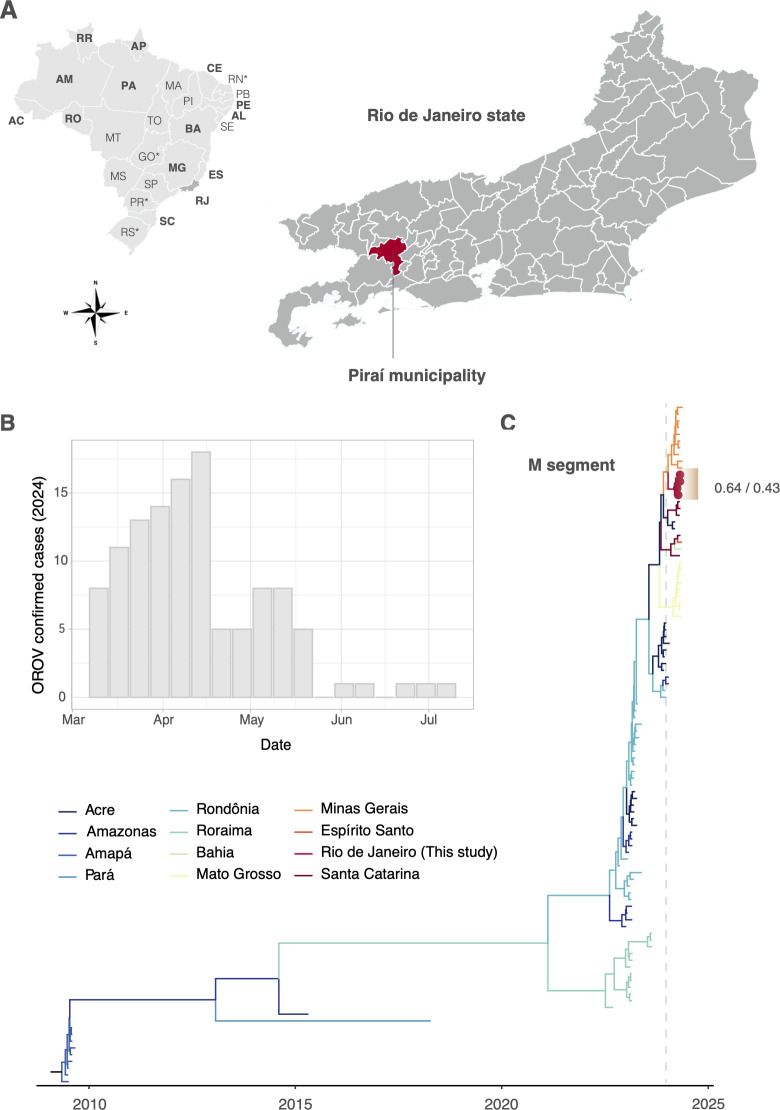
Epidemiological and phylogenetic contextualization of the Oropouche virus outbreak in Rio de Janeiro state, Brazil. (**A**) Map of Brazil and Rio de Janeiro state. Two letter codes for Brazilian states were provided, according to Instituto Brasileiro de Geografia e Estatística (https://www.ibge.gov.br/cidades-e-estados, last accessed 25 October 2024). States with more than 100 OROV cases in 2024 were highlighted with bold fonts. Asterisks denote states where no case was confirmed. The Piraí municipality, marked in dark red, comprises one-third of OROV laboratory-confirmed cases in RJ. (**B**) Number of confirmed OROV infections per epidemiological week in RJ. (**C**) Time-scaled phylogenetic tree of the M segment data set. The model indicates that the sequences cluster in an RJ monophyletic clade, supporting local viral transmission. This clade was dated to the first trimester of 2024 and was most likely introduced from Minas Gerais state. Colors indicate ancestral locations as inferred on a discrete phylogeographic model. Sequences presenting tip shapes (circles) were characterized in this study. Numbers near novel sequences indicate the statistical support for the phylogenetic and trait models, respectively. The dashed gray line marks the beginning of the year 2024.

Prompted by the identification of several cases of OROV throughout Brazil (12), an effort to monitor the initial OROV cases in the state of Rio de Janeiro (RJ) was employed. The State Health Department of RJ initiated a screening effort employing a standardized molecular diagnostic assay (13), revealing 116 RT-qPCR positive samples scattered across the state, with a considerable cluster of cases (*n* = 42, 36.21%) in the small district of Cacaria (Piraí municipality), which has a population of approximately 800 people (Fig. 1A; Fig. S1). Local surveillance detected the first OROV cases in mid-March 2024, with a subsequent increase in cases over the next five epidemiological weeks, followed by a steady decline through early July (Fig. 1B).

To further investigate this outbreak, we performed virus isolation, whole-genome sequencing, and phylogenetic analysis of four clinical samples (see Text S1). Our analyses confirm that the viruses circulating in RJ belong to the reassortant lineage that spread throughout Brazil in 2024 (Fig. S2). The Bayesian phylogenetic reconstruction from the M data set shows that the sequenced isolates form a monophyletic cluster with strong statistical support (posterior probability = 1), dated to February 2024 (95% highest posterior density: 20 January–2 March 2024), indicating a local transmission chain in RJ (Fig. 1C). A discrete phylogeographic reconstruction suggests that the virus was likely introduced from Minas Gerais in early 2024, though with modest statistical support (posterior probability = 0.64; geographic model posterior probability = 0.43). All four isolates obtained from specimens circulating in Cacaria displayed similar plaque morphology and cytopathic effects (CPE) to the prototypic OROV BeAn strain in culture, even though they belong to the reassortant lineage (Fig. S3). As the prototype OROV strain is likely adapted to *in vitro* growth compared to recent isolates, these findings should be interpreted with caution.

This study provides molecular evidence of OROV autochthonous transmission in a non-endemic area. Although the cases were concentrated in Cacaria, this area is linked to larger urban centers, which are well-integrated by road networks to densely populated regions, such as the Rio de Janeiro metropolitan area and the state of São Paulo. Given the local patterns of arbovirus transmission (14), the population’s susceptibility to OROV, and climatic conditions favorable to vectors (15, 16), we hypothesize that the virus could spread further across the state, potentially leading to larger epidemics in future transmission seasons. Continuous epidemiological monitoring through molecular, genomic, and serological methods will be critical to assessing the public health impact of OROV circulation in Rio de Janeiro.

## Supplementary Material

Reviewer comments

## Data Availability

All sequences characterized in this study have been deposited in NCBI GenBank under accession numbers PQ537321–PQ537332. These accession numbers are also listed in Table S1.

## SUPPLEMENTAL MATERIAL

Figure S1 spectrum.02850-24-s0001.tiff Map of Cacaria district (State of Rio de Janeiro).

Figure S2 spectrum.02850-24-s0002.tiff Maximum-likelihood phylogenetic trees inferred from the S, M, and L segment data sets.

Figure S3 spectrum.02850-24-s0003.tif Virological characteristics of OROV clinical isolates.

Supplemental Text spectrum.02850-24-s0004.docx Supplemental methods and legends.

Table S1 spectrum.02850-24-s0005.xlsx Assembly summary statistics and metadata for samples analyzed in this study.

OPEN PEER REVIEW reviewer-comments.pdf An accounting of the reviewer comments and feedback.

ASM does not own the copyrights to Supplemental Material that may be linked to, or accessed through, an article. The authors have granted ASM a non-exclusive, world-wide license to publish the Supplemental Material files. Please contact the corresponding author directly for reuse.
