# Supplementary figures and images for: Genomic evidence of Oropouche virus autochthonous circulation in a small district in the state of Rio de Janeiro, Brazil
Source: Microbiol Spectr. 2025 Feb 11;13(3):e02850-24. doi: 10.1128/spectrum.02850-24 (PMC11878050; doi:10.1128/spectrum.02850-24)

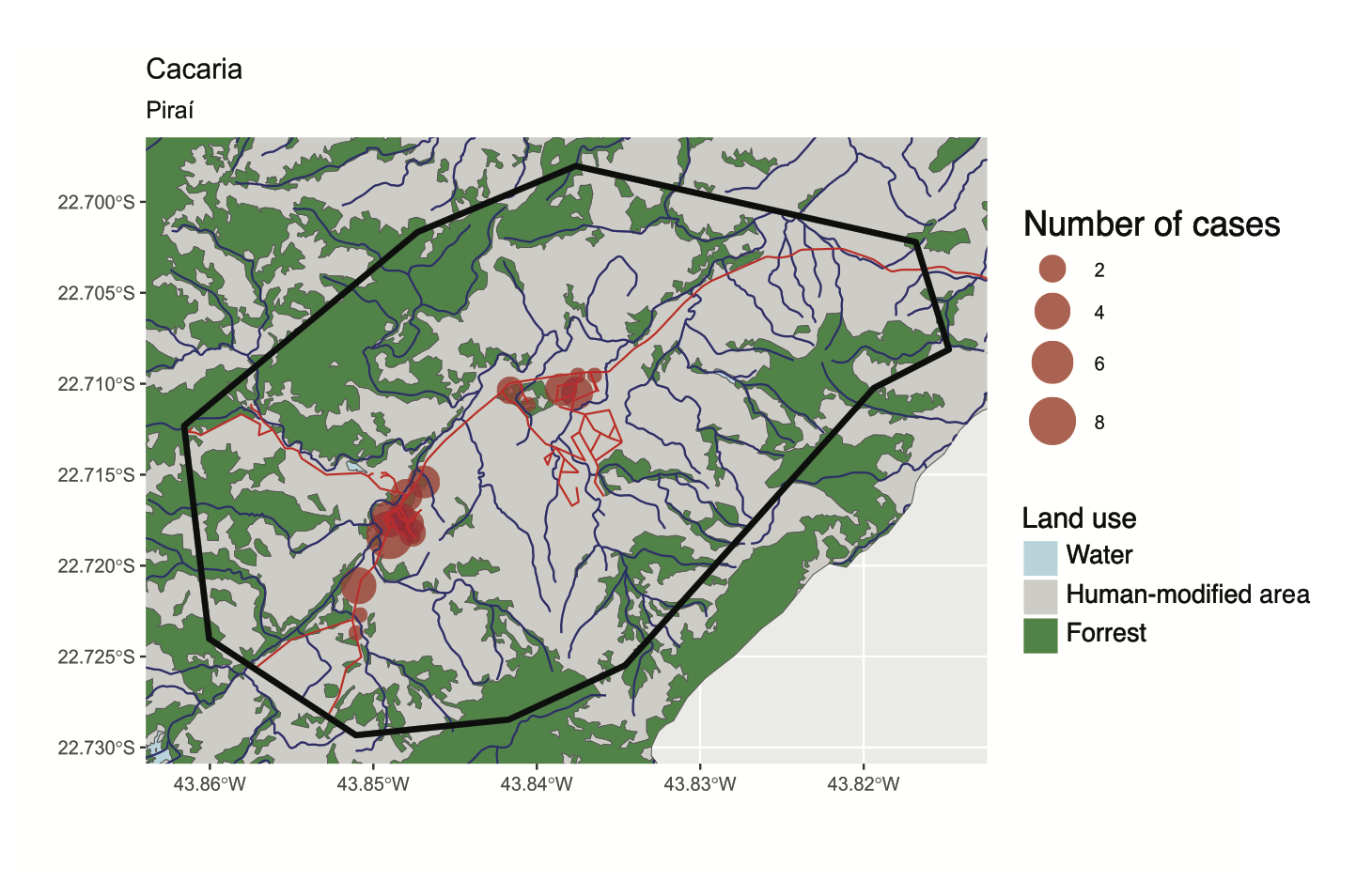

Supplement: Figure S1 — Map of Cacaria district (State of Rio de Janeiro). [file spectrum.02850-24-s0001.tiff]

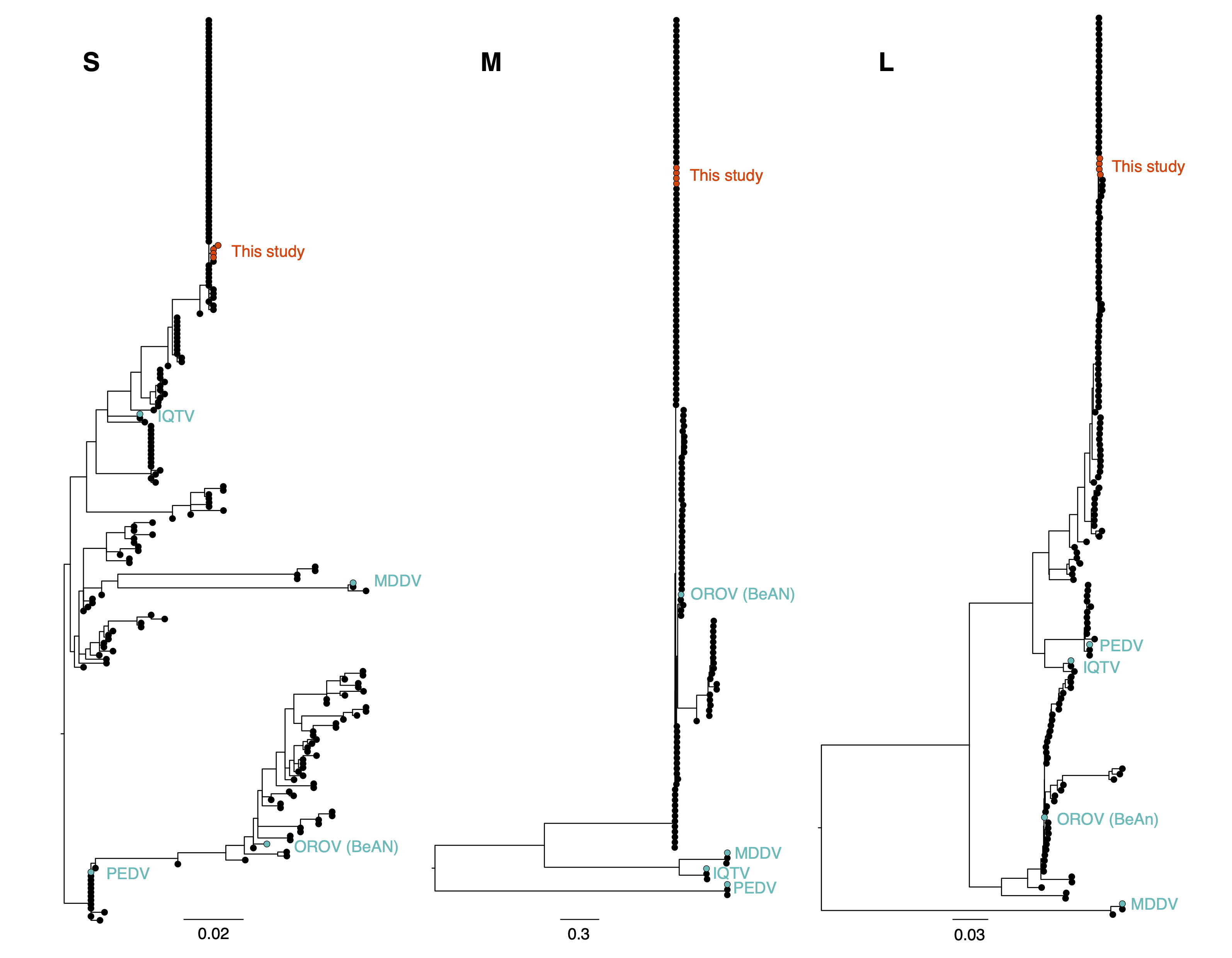

Supplement: Figure S2 — Maximum-likelihood phylogenetic trees inferred from the S, M, and L segment data sets. [file spectrum.02850-24-s0002.tiff]

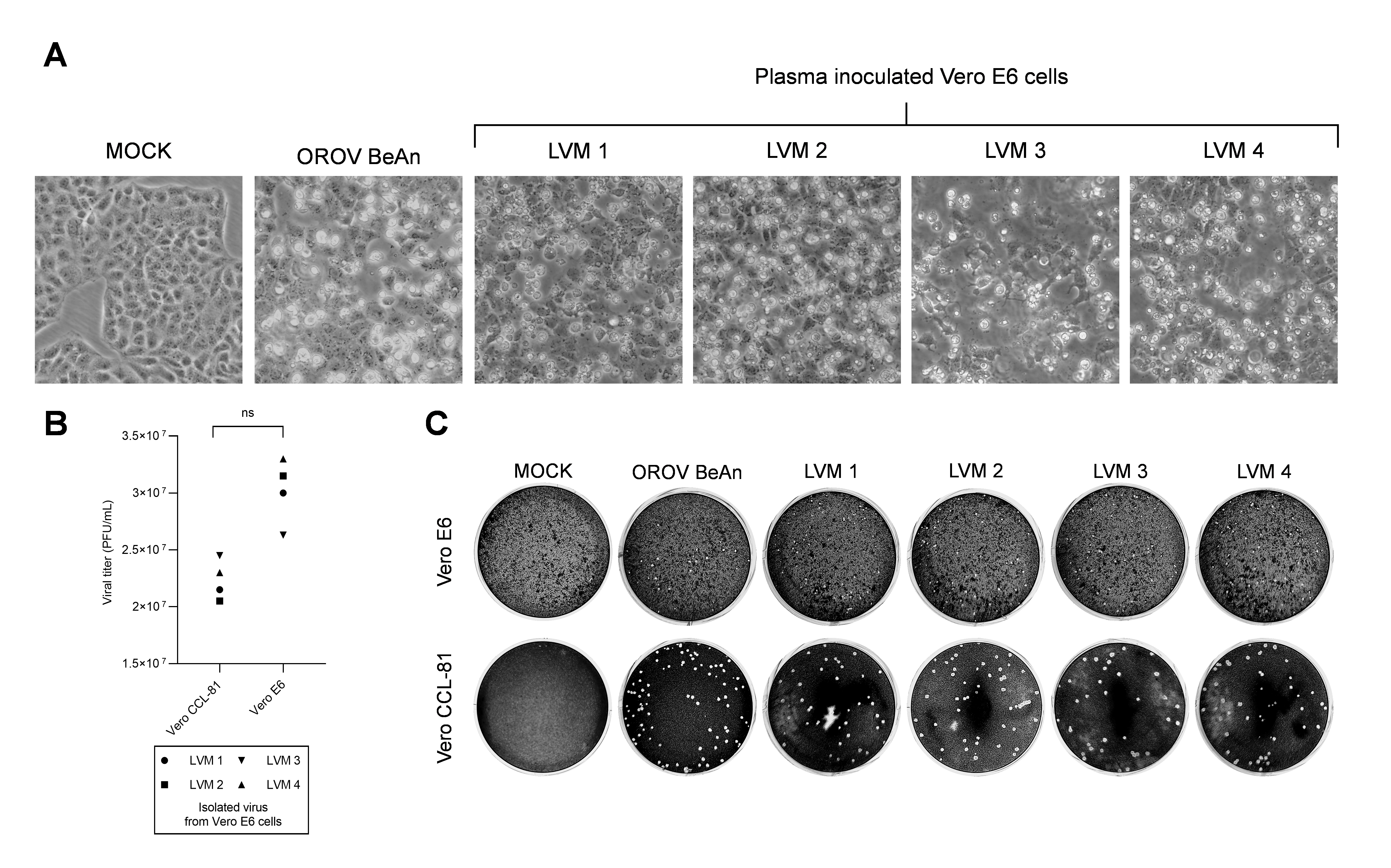

Supplement: Figure S3 — Virological characteristics of OROV clinical isolates. [file spectrum.02850-24-s0003.tif]
