# Supplementary material for: Genomic evidence of Oropouche virus autochthonous circulation in a small district in the state of Rio de Janeiro, Brazil
Source: Microbiol Spectr. 2025 Feb 11;13(3):e02850-24. doi: 10.1128/spectrum.02850-24 (PMC11878050; doi:10.1128/spectrum.02850-24)
Supplement: Supplemental Text — Supplemental methods and legends. [file spectrum.02850-24-s0004.docx]

**Supplementary text**

**1 - Supplementary Methods:**

**1.1 - Epidemiological analysis**

Epidemiological data were retrieved from the online dashboard provided by the Brazilian Ministry of Health (<https://www.gov.br/saude/pt-br/assuntos/saude-de-a-a-z/o/oropouche/painel-epidemiologico>, last accessed 25 October 2024). Additional data from the cases in Piraí municipality were provided by the Rio de Janeiro state Health department (*Secretaria Estadual de Saúde do Estado do Rio de Janeiro*).

**1.2 - Oropouche virus isolation and titration**

All procedures carried out with biological samples and viable Oropouche virus isolates were conducted in the BSL-3 facility from Laboratório de Virologia Molecular (UFRJ). 24h before inoculation, Vero E6 (BCRJ) cells were plated in T75 flasks at a density of 7 x 10^4^ cells/cm^2^ (total of 5.25 x 10^6^ cells) and seeded in DMEM High glucose supplemented with 5% fetal bovine serum (FBS - Gibco). On the day of infection, 100 µL of plasma samples were diluted in 3 mL of DMEM High glucose (Gibco) supplemented with 4% penicillin/streptomycin (Pen/strep - Gibco). Then the suspension was inoculated onto Vero E6 monolayers. After 1h in the incubator (37 °C, atmosphere of 5% CO2), inoculum was removed and 10 mL of DMEM supplemented with 2% FBS and 2% Pen/Strep was added. Cells were monitored daily for cytopathic effect (CPE). At the peak of Vero E6 monolayer disruption, the conditioned medium was harvested, centrifuged at 300 × *g* for 10 minutes, and sterile-filtered in 0.22 µm membrane (JetBiofil) to remove cells and cellular debris. Samples were identified, aliquoted, and stored at −80 °C until titration by plaque assay.

A plaque assay was performed using Vero E6 and Vero CCL-81™ (ATCC) cells to address infectious titers in CPE-positive isolates. Conditioned medium from CPE-positive isolates was serially diluted (10-fold) in DMEM High and inoculated onto confluent cell monolayers seeded in 12-well plates (Costar). After 1h, the inoculum was removed and cells were overlaid with a semisolid medium constituted of alpha-MEM (Gibco) containing 1.5% carboxymethyl cellulose (Sigma Aldrich), 1% FBS and 1% Pen/strep. Cells were further incubated for 4 days after which cells were fixed with 10% formaldehyde. Cells were stained with 0.25% crystal violet in 20% ethanol solution for plaque visualization. Titers were expressed as plaque-forming units (PFU) per milliliter.

**1.3 - Virus whole genome sequencing and assembly**

The isolated viruses were subjected to whole genome sequencing using the multiplex PCR approach [(1)](https://paperpile.com/c/YpsXDP/x2cv), with previously designed pools of primers that generate overlapping amplicons that cover all three segments of the OROV genome, as previously described [(2)](https://paperpile.com/c/YpsXDP/HHYz). Following amplification, libraries were constructed using the COVID-seq library prep kit (Illumina). RNA extraction was performed with the QIAmp viral RNA mini kit (Qiagen). Sequencing was performed on a MiSeq system using a V2 nano cartridge (300 cycles - 2x150). Consensus genome sequences were inferred with minimap v2.17 [(3)](https://paperpile.com/c/YpsXDP/3Blt) and Geneious Prime v2024.0.7. Following a preliminary mapping analysis, segment sequences recently characterized were used as references (NCBI accession numbers - S: PQ066772, M: PQ066778, L: PQ066784) [(2)](https://paperpile.com/c/YpsXDP/HHYz). Sequencing statistics are available in **Table S1**. All genome sequences generated in this study were submitted to NCBI GenBank under accessions PQ537321-PQ537332.

**1.4 - Phylogenetic analyses**

We conducted a series of phylogenetic analyses to contextualize the novel genome sequences. First, we aligned the new S, M, and L sequences to previously published datasets [(2)](https://paperpile.com/c/YpsXDP/HHYz) and performed maximum-likelihood (ML) phylogenetic reconstructions to determine whether these viruses belong to the reassortant lineage driving OROV expansion beyond the Amazon region [(4)](https://paperpile.com/c/YpsXDP/ePN4). Next, we analyzed the novel sequences alongside a broader reference dataset, including all available OROV sequences in NCBI GenBank as of September 2, 2024. Due to gaps in OROV sequence metadata (sampling location and collection date), we manually curated the dataset by searching the scientific literature for missing information. We also filtered out sequences that covered less than 75% of the OROV reference genome, based on NCBI RefSeq (S: NC_005777, M: NC_005775, L: NC_005776). Multiple sequence alignments were performed using MUSCLE v5 [(5)](https://paperpile.com/c/YpsXDP/lEPB), and ML trees were constructed with IQ-Tree v2.2.3 [(6)](https://paperpile.com/c/YpsXDP/xz6j). ModelFinder [(7)](https://paperpile.com/c/YpsXDP/hTsM) was used to identify the best-fitting nucleotide substitution model for each dataset, and SH-aLRT [(8)](https://paperpile.com/c/YpsXDP/V16m) was applied to assess the statistical support of the tree nodes.

To gain further insight into the origins of OROV lineages circulating in RJ, we conducted a Bayesian molecular clock analysis using BEAST v1.10.5 (pre-release v.0.1.2) [(9)](https://paperpile.com/c/YpsXDP/LaDa). This analysis focused on a representative subset of the M dataset, given the higher sequencing coverage for this segment. Building on the previous analysis, this dataset included sequences from a clade associated with the 2023-2024 epidemic, encompassing the new RJ isolates, as well as sequences from various Brazilian states, including regions outside the Amazon. To avoid potential biases in discrete phylogeographic models due to the uneven distribution of sequences across locations, we limited the dataset to a maximum of 10 sequences per Brazilian state. For states with fewer than 10 sequences, all were included; otherwise, sequences were randomly sampled. We also incorporated older external sequences collected as early as 2009 to improve the temporal signal. The final M dataset contained 101 sequences, and its temporal signal was evaluated using a root-to-tip regression, as implemented in TempEst v1.5.3 [(10)](https://paperpile.com/c/YpsXDP/1tDD).

The BEAST model applied the HKY nucleotide substitution model with a parameter for invariable sites [(11)](https://paperpile.com/c/YpsXDP/ULH2), an uncorrelated relaxed clock model with a log-normal distribution of evolutionary rates [(12)](https://paperpile.com/c/YpsXDP/Ydhr), and a non-parametric Skygrid tree prior [(13)](https://paperpile.com/c/YpsXDP/mfz1). We also incorporated the recently proposed Hamiltonian Monte Carlo operator [(14)](https://paperpile.com/c/YpsXDP/fOc0) and two discrete asymmetric phylogeographic models [(15)](https://paperpile.com/c/YpsXDP/rA3h), where model states were defined by either Brazilian federal units (states) or politically defined regions. These analyses were conducted in at least two independent runs of 50 million generations, sampling every 10,000 steps. Mixing and convergence were assessed using Tracer v1.7.2 [(16)](https://paperpile.com/c/YpsXDP/CaNn). Independent chains were merged using LogCombiner, and a maximum clade credibility tree was generated with TreeAnnotator.

**2 - Supplementary Results**

**2.1 - Analysis of Oropouche virus confirmed cases in Cacaria, Piraí municipality, RJ**

We compiled information on confirmed cases in the Cacaria district of Piraí municipality, where 42 out of 116 (36.21%) Oropouche virus infections in Rio de Janeiro state occurred in 2024. Cases were aggregated by residential address (postal code) and plotted in the map, revealing that infections occurred predominantly in areas of higher population density, near local centers, which are connected by roads (Supplementary Figure S1). No pattern related to forest coverage or bodies of water was observed.

**2.2 - Phylogenetic analyses reveal the circulation of a reassortant viral lineage in RJ**

Maximum-likelihood phylogenetic reconstructions of the S, M, and L segments indicate that the newly sequenced viruses cluster within the OROV diversity observed during the 2023–2024 epidemic in Brazil (SH-aLRT > 85 across all segments). These viruses belong to a reassortant lineage, with the M segment being more closely related to the OROV prototype, while the S and L segments cluster within a major clade that is sister to clades encompassing Iquitos virus or Iquitos and Perdões viruses, respectively (Supplementary Figure S2). The root-to-tip regression analysis indicates the M dataset exhibits strong temporal signal (correlation coefficient: 0.89; slope: 7.16 x 10-4).

**2.3 - Oropouche virus was successfully isolated in Vero E6 cells**

To further characterize the OROV RT-PCR positive samples from Cacaria, we established a viral isolation protocol in Vero E6 cells. Surprisingly, all four plasma samples generated positive cytopathic effects (CPE) within 3 to 4 days of infection (**Supplementary Figure S3-A**), which were characterized by cell detachment and nearly complete monolayer disruption in samples LVM-3 and LVM-4. This type of CPE was also exhibited by the OROV BeAn 19991 prototypic strain, used as a control in this study.

Given the abundant CPE observed in all flasks inoculated with plasma samples from patients with OROV fever, we assessed viral titers in the conditioned medium via plaque assay in both Vero E6 and CCL-81 cells. All four samples produced high viral titers, approximately 7 log10 PFU/mL, with minimal variation observed between the cell types tested (**Supplementary Figure S3-B**). However, significant differences were noted in plaque morphology: Vero CCL-81 cells displayed larger, translucent, well-bordered plaques, compared to the smaller, opaque plaques generated in Vero E6 cells. Notably, all plaques generated by the OROV isolates with the novel reassortant genome closely morphologically resembled those observed in the prototypic OROV BeAn strain (**Supplementary** **Figure S3-C**).

**3 - References**

1. [Quick J, Grubaugh ND, Pullan ST, Claro IM, Smith AD, Gangavarapu K, Oliveira G, Robles-Sikisaka R, Rogers TF, Beutler NA, Burton DR, Lewis-Ximenez LL, de Jesus JG, Giovanetti M, Hill SC, Black A, Bedford T, Carroll MW, Nunes M, Alcantara LC, Sabino EC, Baylis SA, Faria NR, Loose M, Simpson JT, Pybus OG, Andersen KG, Loman NJ. 2017. Multiplex PCR method for MinION and Illumina sequencing of Zika and other virus genomes directly from clinical samples. *Nat Protoc* 12:1261–1276.](http://paperpile.com/b/YpsXDP/x2cv)

2. [Moreira FRR, Dutra JVR, de Carvalho AHB, Reis CR, Rios JSH, de Oliveira Ribeiro M, Arruda MB, Alvarez P, Souza RP, Voloch C, Zauli DAG, Aguiar RS. 2024. Oropouche virus genomic surveillance in Brazil.](http://paperpile.com/b/YpsXDP/HHYz) [Lancet Infect Dis 24(11): e664-e666.](http://paperpile.com/b/6BuwCQ/104dm)

3. [Li H. 2021. New strategies to improve minimap2 alignment accuracy. *Bioinformatics* 37:4572–4574.](http://paperpile.com/b/YpsXDP/3Blt)

4. [Naveca FG, de Almeida TAP, Souza V, Nascimento V, Silva D, Nascimento F, Mejía M, de Oliveira YS, Rocha L, Xavier N, Lopes J, Maito R, Meneses C, Amorim T, Fé L, Camelo FS, de Aguiar Silva SC, de Melo AX, Fernandes LG, de Oliveira MAA, Arcanjo AR, Araújo G, André Júnior W, de Carvalho RLC, Rodrigues R, Albuquerque S, Mattos C, Silva C, Linhares A, Rodrigues T, Mariscal F, Morais MA, Presibella MM, Marques NFQ, Paiva A, Ribeiro K, Vieira D, da Silva Queiroz JA, Passos-Silva AM, Abdalla L, Santos JH, de Figueiredo RMP, Cruz ACR, Casseb LN, Chiang JO, Frutuoso LV, Rossi A, Freitas L, de Lima Campos T, Wallau GL, Moreira E, Lins Neto RD, Alexander LW, Sun Y, de Filippis AMB, Gräf T, Arantes I, Bento AI, Delatorre E, Bello G. 2024. Human outbreaks of a novel reassortant Oropouche virus in the Brazilian Amazon region. *Nat Med*](http://paperpile.com/b/YpsXDP/ePN4) [30:3509–3521.](http://paperpile.com/b/6BuwCQ/WgTcP)[.](http://paperpile.com/b/YpsXDP/ePN4)

5. [Edgar RC. 2022. Muscle5: High-accuracy alignment ensembles enable unbiased assessments of sequence homology and phylogeny. *Nat Commun* 13:1–9.](http://paperpile.com/b/YpsXDP/lEPB)

6. [Minh BQ, Schmidt HA, Chernomor O, Schrempf D, Woodhams MD, von Haeseler A, Lanfear R. 2020. IQ-TREE 2: New Models and Efficient Methods for Phylogenetic Inference in the Genomic Era. *Mol Biol Evol* 37:1530–1534.](http://paperpile.com/b/YpsXDP/xz6j)

7. [Kalyaanamoorthy S, Minh BQ, Wong TKF, von Haeseler A, Jermiin LS. 2017. ModelFinder: fast model selection for accurate phylogenetic estimates. *Nat Methods* 14:587–589.](http://paperpile.com/b/YpsXDP/hTsM)

8. [Guindon S, Dufayard J-F, Lefort V, Anisimova M, Hordijk W, Gascuel O. 2010. New Algorithms and Methods to Estimate Maximum-Likelihood Phylogenies: Assessing the Performance of PhyML 3.0. *Syst Biol* 59:307–321.](http://paperpile.com/b/YpsXDP/V16m)

9. [Suchard MA, Lemey P, Baele G, Ayres DL, Drummond AJ, Rambaut A. 2018. Bayesian phylogenetic and phylodynamic data integration using BEAST 1.10. *Virus Evol* 4:vey016.](http://paperpile.com/b/YpsXDP/LaDa)

10. [Rambaut A, Lam TT, Max Carvalho L, Pybus OG. 2016. Exploring the temporal structure of heterochronous sequences using TempEst (formerly Path-O-Gen). *Virus Evol* 2:vew007.](http://paperpile.com/b/YpsXDP/1tDD)

11. [Hasegawa M, Kishino H, Yano T. 1985. Dating of the human-ape splitting by a molecular clock of mitochondrial DNA. *J Mol Evol* 22.](http://paperpile.com/b/YpsXDP/ULH2)

12. [Drummond AJ, Ho SYW, Phillips MJ, Rambaut A. 2006. Relaxed Phylogenetics and Dating with Confidence. *PLoS Biol* 4:e88.](http://paperpile.com/b/YpsXDP/Ydhr)

13. [Gill MS, Lemey P, Faria NR, Rambaut A, Shapiro B, Suchard MA. 2012. Improving Bayesian Population Dynamics Inference: A Coalescent-Based Model for Multiple Loci. *Mol Biol Evol* 30:713–724.](http://paperpile.com/b/YpsXDP/mfz1)

14. [Baele G, Gill MS, Lemey P, Suchard MA. 2020. Hamiltonian Monte Carlo sampling to estimate past population dynamics using the skygrid coalescent model in a Bayesian phylogenetics framework. *Wellcome Open Res* 5:53.](http://paperpile.com/b/YpsXDP/fOc0)

15. [Lemey P, Rambaut A, Drummond AJ, Suchard MA. 2009. Bayesian Phylogeography Finds Its Roots. *PLoS Comput Biol* 5:e1000520.](http://paperpile.com/b/YpsXDP/rA3h)

16. [Rambaut A, Drummond AJ, Xie D, Baele G, Suchard MA. 2018. Posterior Summarization in Bayesian Phylogenetics Using Tracer 1.7. *Syst Biol* 67:901–904.](http://paperpile.com/b/YpsXDP/CaNn)

**Figure Legend:**

**Supplementary Figure S1. Map of Cacaria district (State of Rio de Janeiro).** The map depicts the small district of Cacaria, which harbored a significant number of OROV cases in Rio de Janeiro state. Circles in red pinpoints aggregated confirmed cases by neighborhood. The red line indicates roads. Surface colors indicate land usage patterns (green: forest areas, grey: human-modified space, blue: water). This map highlights the spatial aggregation of cases, supporting the implementation of entomological surveillance in impacted areas.

Supplementary **Figure S2. Maximum-likelihood phylogenetic trees inferred from the S, M, and L segments datasets.** These phylogenetic reconstructions indicate that the new viruses cluster with sequences from 2023-2024, displaying the same reassortant pattern observed in recent epidemics in northern Brazil, which is spreading across the country. These viruses harbor segment M more closely related to the OROV (BeAn) prototype strain, while the S and L segments are more closely related to alternative prototypical lineages, such as the Iquitos virus and Perdões virus. Tip shapes colored in red and blue represent new and prototypical virus sequences, respectively. OROV: Oropouche virus (BeAn prototypical strain); IQTV: Iquitos virus; PEDV: Perdões virus; MDDV: Madre de Dios virus. The scale indicates nucleotide substitutions per site.

**Supplementary Figure S3. Virological characteristics of OROV clinical isolates -** (**A**) Visualization of the cytopathic effect (CPE) in Vero E6 cells inoculated with plasma samples from identified subjects after three days of incubation. Massive cell detachment was observed in all plasma-inoculated flasks. (**B**) Comparison of viral titers obtained from Vero E6 and Vero CCL-81 cells used for plaque assays to determine the viral infectivity of CPE-positive Vero E6 cells supernatants after inoculation. Data expressed as plaque-forming units per milliliter (PFU/mL). Statistical analysis was performed by two-way ANOVA using Tukey's test for multiple comparisons. (**C**) Visualization of plaque formation in Vero E6 cells (upper panel) and Vero CCL-81 cells (lower panel). Images are from representative wells used for the plaque count shown in graph (B).

**Table Legend:**

**Supplementary Table S1. Assembly summary statistics and metadata for samples analyzed in this study.** Metadata presented are location and collection data for the four samples analyzed in this study. Assembly summary statistics include the number of reads generated for each sample and the Genbank accession number for each genome segment. The number of mapped reads and the percentage of coverage refer to the reference genomes of each of the genomic fragments (NCBI accession numbers - S: PQ066772, M: PQ066778, L: PQ066784).
